# Amalgam Filling

**Published:** 1892-08

**Authors:** R. Ottolengui


ARTICLE IX.

### AMALGAM FILLING.

---

It is frequently admitted that amalgam has been a much-abused material. This charge against dentists is more true in relation to contour fillings than in any other connection. It will not suffice to say that, for contouring, amalgam, is a *valuable* agent. It is necessary to say that it is *invaluable*. Its usefulness is inestimable. It may be made to save teeth which, without it, would be lost, or, at least, even if saved, would be of but slight service for mastication.

In the realm of contour work, amalgam occupies a place that is unique. With it can be restored all those forlorn cases, those wrecks, which half a century ago were inevitably consigned to the forceps. Yet, with shame it must be admitted that only a very few men know how to obtain the most desirable results with amalgam in these very cases. The man who can restore a molar where caries has advanced beneath the gum, two or three cusps being entirely absent, and build on this unpromising foundation a tooth which becomes as useful as the original, and which, moreover, remains permanently in place without fretting the gum and without inviting decay along its borders, has more right to count himself skilled than the best gold-filler.

In small cavities the plastic is the more manageable material, but as the size of a cavity increases, manipulation with gold becomes *less* difficult, the added obstacles being only the tediousness of a lengthy sitting. With amalgam it is otherwise, for the larger the cavity the *more* difficult it becomes to attain the highest success.

Amalgam, then, in contour work may well attract our special attention. I must point out the obstacles to its proper use, and tell how to combat them. How often have we all expended a half hour or more restoring a lost corner of a molar, only to have it return on the following day, with a portion missing? We say to the patient. "You must have bitten something on that before it was thoroughly hardened," but that is no satisfaction either to patient or dentist.

This tendency to fracture, in an amalgam filling, is due to several things. Of course, if the occlusion be sharp, the explanation given by the dentist may be true; mastication may have dislodged a portion of the mass. But where such an accident is possible, the dentist must note the fact and guard against it in advance. The filling must leave him so shaped that it will not be disturbed by the closing of the jaws, even so much as by the production of a slight scratch, by the perpendicular or lateral action of the jaw. The patient must be asked to gently move the jaw from side to side, as he would do in eating. This brings the cusps of the opposing teeth into all the different relations which they are to bear, and if the filling is unmarred by this, the single warning to chew on the opposite side during the succeeding day, if obeyed, will bring the filling in good condition, or we must protect the filling, temporarily building a tooth with gutta-percha.

But it is often by a cause other than mastication that the filling is broken. We take the utmost care to keep a gold filling free from moisture, yet some men do not hesitate to insert amalgam with the cavity and surrounding parts flooded with saliva. This so-called submarine work not only should not be practiced, and a clinician showing it before assembled students, or practitioners, should be roundly condemned. In contour work with amalgam it is of the utmost importance that the perfect crystallization of the mass should not be interfered with by moisture. The filling should be kept dry throughout the whole operation, if possible. Where the cavity extends far beneath the gum-margin, the tooth may be filled in two operations, though at the same sitting. Using the napkin as a dam, amalgam must be packed till it extends beyond the margin, sufficiently far so that the rubber-dam may be placed. Then the cavity, and the amalgam already in place, may be dried and the filling continued. Thus it is shown that because a cavity cannot be kept dry with rubber-dam from the out-set, that is no reason why the filling should be allowed to become inundated several times, through a vain effort to control moisture with a napkin.

The next important point is to avoid fracturing the mass during the operation. This involves simply the proper application of force, and the proper consistency of the material. Amalgam, for use in a large contour, must be prepared slightly more plastic than for ordinary work. It is to be packed with balls of bibulous paper, with a wiping motion, thus forcing the material against the cavity-walls, as long as this action can be carried out. By this course the excess of mercury is forced out, and crystallization begins at once. Thenceforth the particles of that portion of the mass already in position *must not be disturbed.* If, by exerting force in a wrong direction, a part of the filling is fractured off, *it is folly to hope to get a good result by patting it back with a burnisher. The re-union will not be as strong as was the original union.* It would be better, where the accident does occur, to remove the separated piece, replacing it with freshly-mixed material.

Whether the remainder of the filling be packed with bibulous paper or with burnisher, it is from this point on that fracture is to be feared. The rule is very simple. *Pressure must be exerted only in line with the greatest resistance, offered by the tooth itself.*

The last essential is to dismiss the filling as far advanced toward crystallization as possible. This may be best accomplished by burnishing gold into it.—R. OTTOLENGUI, in *Cosmos.*

